# The Temporal Winner-Take-All Readout

**DOI:** 10.1371/journal.pcbi.1000286

**Published:** 2009-02-20

**Authors:** Maoz Shamir

**Affiliations:** Department of Physiology and the Zlotowski Center for Neuroscience, Ben-Gurion University of the Negev, Beer-Sheva, Israel; University College London, United Kingdom

## Abstract

How can the central nervous system make accurate decisions about external stimuli at short times on the basis of the noisy responses of nerve cell populations? It has been suggested that spike time latency is the source of fast decisions. Here, we propose a simple and fast readout mechanism, the temporal Winner-Take-All (tWTA), and undertake a study of its accuracy. The tWTA is studied in the framework of a statistical model for the dynamic response of a nerve cell population to an external stimulus. Each cell is characterized by a preferred stimulus, a unique value of the external stimulus for which it responds fastest. The tWTA estimate for the stimulus is the preferred stimulus of the cell that fired the first spike in the entire population. We then pose the questions: How accurate is the tWTA readout? What are the parameters that govern this accuracy? What are the effects of noise correlations and baseline firing? We find that tWTA sensitivity to the stimulus grows algebraically fast with the number of cells in the population, *N*, in contrast to the logarithmic slow scaling of the conventional rate-WTA sensitivity with *N*. Noise correlations in first-spike times of different cells can limit the accuracy of the tWTA readout, even in the limit of large *N*, similar to the effect that has been observed in population coding theory. We show that baseline firing also has a detrimental effect on tWTA accuracy. We suggest a generalization of the tWTA, the *n*-tWTA, which estimates the stimulus by the identity of the group of cells firing the first *n* spikes and show how this simple generalization can overcome the detrimental effect of baseline firing. Thus, the tWTA can provide fast and accurate responses discriminating between a small number of alternatives. High accuracy in estimation of a continuous stimulus can be obtained using the *n*-tWTA.

## Author Summary

Considerable experimental as well as theoretical effort has been devoted to the investigation of the neural code. The traditional approach has been to study the information content of the total neural spike count during a long period of time. However, in many cases, the central nervous system is required to estimate the external stimulus at much shorter times. What readout mechanism could account for such fast decisions? We suggest a readout mechanism that estimates the external stimulus by the first spike in the population, the tWTA. We show that the tWTA can account for accurate discriminations between a small number of choices. We find that the accuracy of the tWTA is limited by the neuronal baseline firing. We further find that, due to baseline firing, the single first spike does not encode sufficient information for estimating a continuous variable, such as the direction of motion of a visual stimulus, with fine resolution. In such cases, fast and accurate decisions can be obtained by a generalization of the tWTA to a readout that estimates the stimulus by the first *n* spikes fire by the population, where *n* is larger than the mean number of baseline spikes in the population.

## Introduction

In recent years, there has been growing interest in coding information about external stimuli by the fine temporal structure of the neural dynamic response [1]–[18]. Several studies have shown that response latency is modulated by external stimuli [1]–[4]. Many cells in the middle temporal (MT) cortex code for the direction of motion of visual stimuli, and can be characterized by a preferred direction of the stimulus, to which they respond maximally, see e.g., [19],[20]. Osborne et al. [1] reported that the MT cells respond with the shortest delay when the stimulus is in their preferred direction and that the response delay increases as the stimulus direction diverges from the preferred direction of the cell. In the auditory system of the ferret, Nelken et al. [2] showed response-latency tuning in primary auditory cortex cells to the direction of a virtual sound source. In a recent work Gollisch and Meister [18] showed that relative first-spike times of retinal ganglion cells carry considerable information about the external stimulus, but they did not suggest a concrete readout mechanism.

Here we study the accuracy of a simple readout mechanism, the temporal-Winner-Take-All (tWTA), which extracts information from response latency. The tWTA estimates the stimulus by the identity of the cell that fired the *first* spike in a population of cells, in contrast to the conventional rate-Winner-Take-All (WTA), which estimates the stimulus by the identity of the cell that fired the *most* spikes. For example, the tWTA estimate for the direction of motion of a visual stimulus from the responses of a population of MT cells would be the preferred direction of the cell that fired the first spike in the entire population.

Considerable theoretical effort has been devoted to the study of the accuracy of population code readout mechanisms, such as the population-vector, optimal-linear and ideal observer readouts. Of particular interest in the investigation of these readouts was the dependence of the readout accuracy on the population size and the effects of noise correlations in the neuronal responses. In this work, we quantify tWTA accuracy. To this end, we address three specific questions. One, what are the essential features of the neuronal dynamic response to the stimulus to which the tWTA is sensitive? Two, how does the tWTA accuracy depend on the population size? Three, what are the effects of noise correlations and baseline firing on tWTA accuracy?

These questions are addressed in the framework of a statistical model for the dynamic response of MT cells to a moving visual stimulus. In the first part of the results section we investigate tWTA accuracy in a two-column competition model, and in the second part we study tWTA accuracy in the framework of a hypercolumn model. Both parts start by defining the statistical model of the neuronal dynamic response and then follow with an investigation of tWTA accuracy in the absence of noise correlations and baseline firing. In the final stage of each part, correlations and baseline firing are introduced and their effect on tWTA accuracy is investigated.

## Results

### tWTA Readout Accuracy in a Two Competing Columns Model

#### The model

We study tWTA accuracy in a model of two competing MT columns coding for the direction of motion of visual stimuli. Each column is comprised of 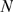 homogeneous cells. We denote the preferred direction of the cells in column 1 by 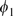 and the preferred direction of the cells in column 2 by 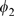. Without loss of generality, we take 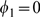, which is equivalent to measuring all angles with respect to 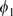. We denote the probability density of a single cell 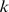 (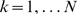) in column 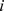 with preferred direction 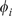 to fire its first spike at time 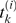 given that stimulus 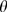 was presented at time 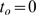 by 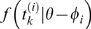. Assuming that first-spike times are statistically independent, the probability density of the first spike in the entire column 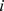 at time 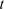 is given by the product of three terms: the probability density of a specific cell to fire its first at time 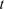, 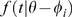, the probability that that the first spike times of the rest 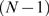 cells in the population occurred after time *t*, 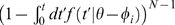, and the 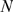 different possibilities of choosing the cell that fired the first spike:
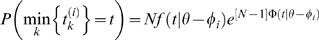
(1)

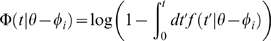
(2)The function 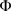 is the logarithm of the probability of a single cell firing its first spike after time 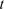, and it has the following properties: 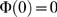, 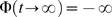 and 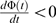. Equation (1) can also be obtained by taking the derivative of the probability that the first spike in the column occurred after time 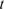: 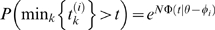, with respect to first spike time, 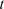.

Throughout this section, we will quantify tWTA accuracy by using the two alternative forced choice (2AFC) paradigm. In a 2AFC discrimination task, the system is given a stimulus, either 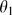 or 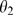, randomly with equal probabilities. Presentation of the stimulus generates a population response in the two columns, 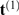 and 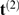, which are distributed as defined above. The task of the readout is to infer, on the basis of these spike times, whether the stimulus was 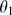 or 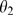. We will use the probability of correct discrimination, 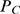, and the error rate, 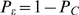, as measures of the tWTA performance. We will use the term *sensitivity* to designate the inverse of the stimulus difference, 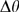, at which 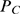 crosses a certain threshold, 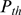. This latter measure is related to the ‘*just noticeable difference*’ used in psychophysics.

#### tWTA accuracy in the absence of correlations

Assuming that column 1 responds faster to a stimulus in direction 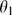 than column 2 and vice versa for stimulus 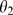, we define the tWTA readout in the 2AFC task as follows:
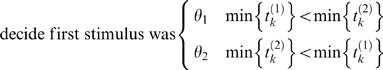
(3)For the sake of convenience, we take 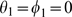 and 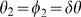. This choice equalizes the probability of correct response given stimulus 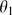 and given stimulus 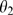. The probability of correct response, 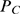, is given by the probability that population 1 fired the first spike, given stimulus 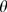. Thus, 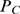 can be written as the integration over all possible first-spike times, 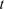, of the probability density that population 1 fired its first spike at time 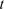 multiplied by the probability that the first spike time of population 2 is large than 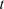:
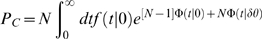
(4)In the limit of large populations, 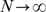, the integral in the right-hand-side of equation (4) will be dominated by the region in which the exponent obtains its maximum. Since 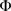 is a monotonically decreasing function of 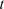, this region is the region of small 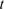. For small 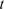, we approximate 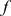 by:
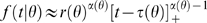
(5)where 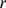 is the scale parameter, 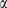 is the shape parameter, 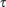 is the delay parameter and 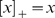 for 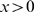 and 0 otherwise.

#### Relation to the peri stimulus time histogram (PSTH) in an inhomogeneous Poisson process (IHPP)

**Figure 1:**
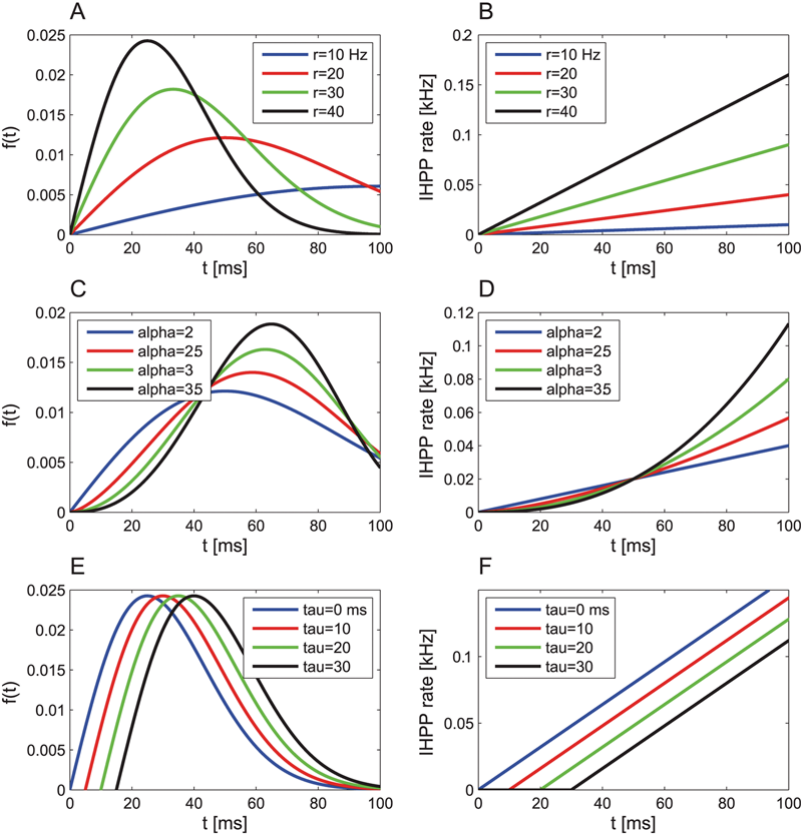
Three examples showing the effects of the scale parameter (a,b), the shape parameter (c,d), and the delay parameter (e,f) on the first spike time probability density, 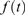, (left column) and the PSTH rate, 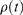, of a corresponding inhomogeneous Poisson process (right column). The PSTHs were taken to be of the form of 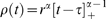 (compare with equation 5), and 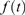 is obtained via the relation of equation (6). The parameters used to generate the plots are as follows. For a and b: 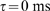, 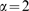, and 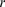 as appears on the figure. For (c,d): 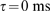, 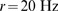, and 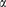 as appears on the figure. For (e,f): 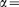, 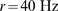, and 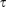 as appears on the figure.

The IHPP is widely used to model the stochastic nature of the neural temporal response [21],[22] and is fully defined by the PSTH. In the context of first spike-time distribution, the choice of an IHPP model does not limit the generality of the model, since every PSTH, 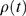, of an IHPP could be mapped to first spike time distribution, 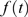, and vice versa. For a given IHPP with PSTH, 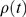, the first spike time distribution is given by (see e.g., [21],[22])
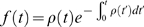
(6)In the other direction, we want to obtain the PSTH, 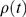, that will yield a specific first spike time distribution, 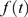, in an IHPP model. The probability density that the first spike has occurred in time 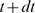 in an IHPP model, can be written as the product of the probability density of spiking at that time, 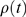, multiplied by the probability that there were no prior spikes, 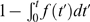; hence, 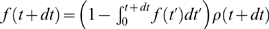. Thus we obtain the reciprocal relation
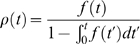
(7)which could be verified by substituting equation (7) into equation (6). For small 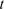: 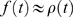. Thus, the scale parameter corresponds to the scale of the PSTH, the shape parameter governs the initial acceleration of the PSTH, and the delay parameter measures the temporal shift of the PSTH. Figure 1 illustrates how the different parameters that characterize the initial neural response: scale, shape and delay, affect the first spike probability density and the corresponding PSTH. Note that whereas 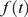 and 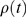 are very similar for small 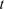, for large 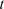, 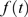 decays to zero while 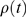 may continue to increase. Below we study the different effects of the tuning of these parameters on the accuracy of the tWTA.

#### Effect of scale parameter tuning

**Figure 2:**
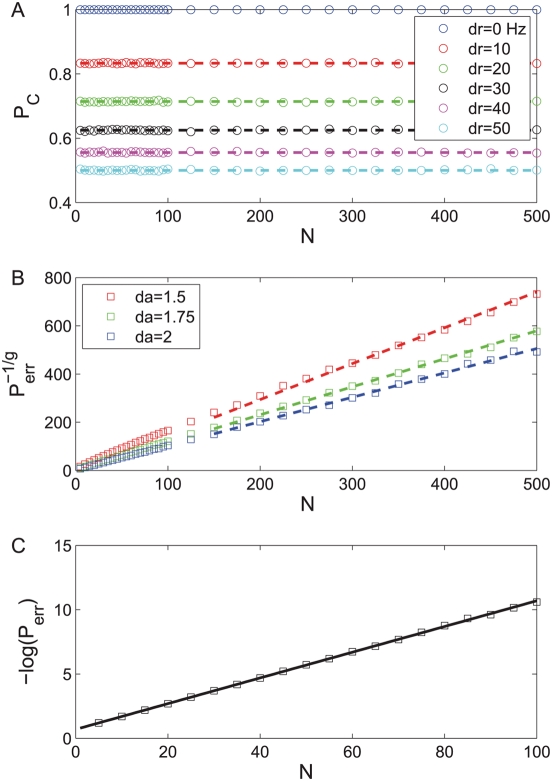
tWTA performance in a 2AFC discrimination task between stimulus 0° and 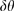 in a two-column model as function of the number of cells in the population. Open symbols show numerical estimation of the tWTA performance as obtained by averaging the probability of correct discrimination over 10^6^ realizations of the stochastic neural responses. Probability distribution of first spike times followed an IHPP with the following PSTHs. (a) Scale parameter tuning: 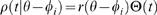 with 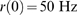 and 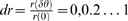 from top to bottom. The dashed lines show the analytical prediction of equation (9). (b) Shape parameter tuning: 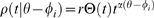 with 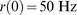, 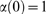 and 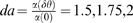 from top to bottom. The tWTA performance is shown in terms of 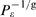 where 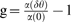. The dashed lines show linear regression lines in keeping with the prediction of equation (12). (c) Delay parameter tuning: 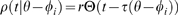 with 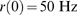, 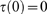 and 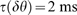. The tWTA performance is shown in terms of minus the log of the error rate. The solid line shows the analytical prediction of equation (15).

We first consider a simple model in which the scale is the only parameter that is tuned to the stimulus. In this case, we can write 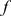 near 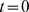 as the product of a function of the stimulus and a function of time:
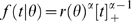
(8)where 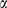 is independent of 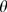. Expanding 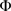 in small 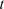, 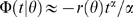 and substituting in equation (4), we obtain to a leading order in 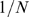


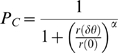
(9)Hence, in this case, the probability of correct response is at chance level, 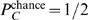, when the neural response has the same scale for the two alternatives, 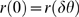, and increases monotonically in the ratio 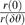. The accuracy of the tWTA is not improved by increasing 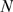: The same accuracy will be obtained with 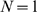 and 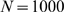 cells, but, somewhat faster for the 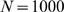 case. Figure 2a shows the probability of correct discrimination as a function of 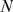 for different values of 
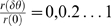
 from top to bottom. The open circles are estimates of the tWTA accuracy obtained by averaging the tWTA accuracy over 10^6^ realizations of the neural stochastic response. The dashed line shows the analytical prediction of equation 9 with 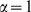.

#### Effect of shape parameter tuning

In the case where only the shape parameter, 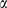, is tuned to the stimulus, we write:
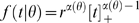
(10)where 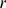 is independent of 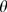. We assume that population 1, with preferred direction 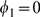, fires faster than population 2, with preferred direction 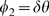, given stimulus 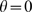, in the sense that for short times the probability of firing of cell in population 2 is larger than that in population 1; hence, 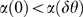. To compute 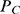 in the limit of large populations, equation (4), it is convenient to make a change of variables to 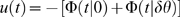, yielding:
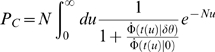
(11)where 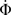 is the time derivative of 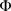, 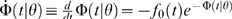. To leading order in small 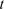 for 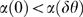, 
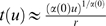
. Applying Watson's Lemma [23] we obtain the asymptotic approximation for the error rate:
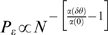
(12)Hence, in this case, the probability of error decays algebraically with 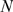 to zero. This scaling of the readout accuracy with population size is similar to the scaling of the conventional rate-WTA accuracy with population size [24]. For small 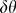, 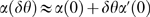, we obtain:
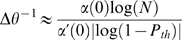
(13)Thus, although in this case tWTA sensitivity improves by utilizing larger populations, this logarithmic improvement is extremely slow. Figure 2b shows the discrimination error rate to the power of 
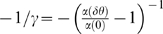
 as a function of 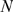 for different values of 
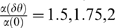
 from top to bottom. The open squares are estimates of the tWTA accuracy obtained by averaging tWTA accuracy over 10^6^ realizations of the neural stochastic response. The dashed lines show linear regression fits to the curves, in keeping with the asymptotic relation of equation (12).

#### Effect of delay parameter tuning

In the case where the delay parameter, 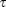, is the only the parameter that is tuned to the stimulus, we write:
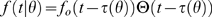
(14)where 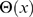 is the Heavyside function: 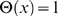 for 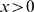 and 0 otherwise. In this case. we find (see Methods) that the probability of error decays exponentially fast with the population size, 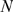:
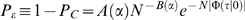
(15)

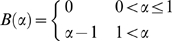
(16)where 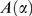 is defined in Methods. Hence, in this case, the tWTA error rate decays to zero exponentially with 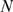, in contrast to the slow algebraic scaling of the conventional rate-WTA accuracy with the population size [24]. For small 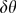, we can expand the delay parameter, 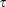, in 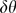 and approximate 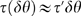; for small 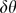, we thus find that tWTA sensitivity grows algebraically with 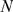:
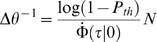
(17)in contrast to the logarithmic scaling of the conventional rate-WTA sensitivity with population size [24]. Figure 2c shows minus the logarithm of the discrimination error rate in the case of delay parameter tuning to the stimulus. The open squares are estimates of the tWTA accuracy obtained by averaging tWTA accuracy over 10^6^ realizations of the neural stochastic response. The solid line shows the analytical prediction of equation (15).

The different effects exerted by scale, shape and delay parameters on the scaling of the tWTA accuracy with the population size highlights the sensitivity of the tWTA to fine details of the first-spike-time distribution. Nevertheless, in the general case, all parameters will be tuned to the stimulus. The dominant contribution to the tWTA accuracy will result from the tuning of the delay parameter. Hence, the tWTA error rate will decay exponentially fast to zero with 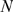, and the sensitivity will scale algebraically with 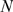. We will therefore focus hereafter on models in which the delay parameter is tuned to the stimulus and ignore the tuning of other parameters to the stimulus.

Two important factors may have a considerable effect on the tWTA accuracy are addressed below. The first is noise correlations in the fluctuations of first spike times of different cells. It has been shown that noise correlations have a considerable effect on population code readout accuracy [25]–[29]. The second factors is nonzero baseline firing rate.

#### Effect of correlations on the tWTA accuracy

**Figure 3:**
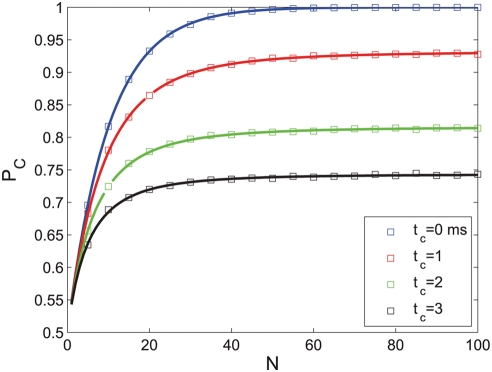
Effect of correlations on the tWTA readout accuracy. The probability of tWTA correct response, 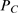, in the presence of noise correlations is shown as a function of the population size, 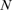. Open squares show numerical estimation of the probability of correct response by averaging over 10^5^ trials of simulating the network stochastic response. The model was defined as in section ‘effect of correlations on the tWTA accuracy’. We write the first spike time 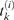 of neuron 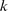 in population 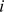 as the sum of a correlated term and an independent term: 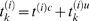 (see equation 18), where 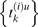 are statistically independent, given the stimulus, with probability distribution 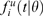. Specifically, here we took: 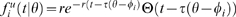 with 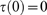 and 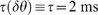. The probability density of the correlated part, 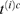, is given by 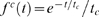. The parameters that were used for the simulations are: 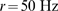, 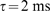 and 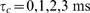 from top to bottom. The solid lines show the analytical result of equation (20).

How should the covariance between first spike times of different cells be modeled? One possible mechanism that can cause correlated firing is having a shared input. Two cells that receive a common input that fluctuates above its mean will integrate it over time and reach spiking threshold sooner than their average first spike time. If the common input fluctuates below its average value, spike time of both cells will be delayed. It is reasonable to assume that cells that are *functionally* close, i.e., have similar preferred directions, will have more common input. Hence, their first spike times are expected to be more positively correlated. motivated by this intuition, we model correlations by adding a uniform random shift, 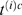, to the spike times of the cells in column 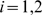, which represents the effect of fluctuations in shared inputs to cells in every column. Thus, we write the first spike time 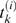 of neuron 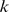 in population 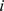 as the sum of a correlated term and an independent term:
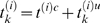
(18)where 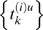 are statistically independent, given the stimulus, with probability distribution 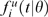. We assume that, given stimulus 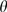, 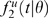 is delayed relative to 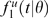 by 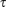, i.e., 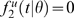 for 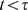 whereas 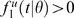 for 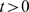. The correlated components, 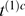 and 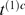, are independent, with probability distribution 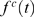. In the limit of large 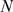, the probability of correct discrimination is given by (see Methods):
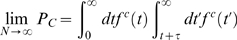
(19)Hence, for large populations, the uncorrelated fluctuations can be ignored, and the probability of correct discrimination saturates to a size-independent limit. Figure 3 shows the performance of the tWTA, in terms of percent correct discrimination, as a function of the number of cells in each column, 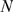, for increasing values of 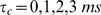 from top to bottom. In the simulations, we used a model in which only the delay parameter is tuned to the stimulus. Specifically we took: 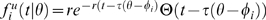 with 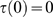 and 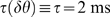. For the correlated, part we used 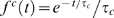. In this case we obtain (see Methods):
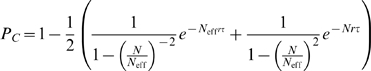
(20)

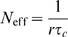
(21)In the absence of correlations, 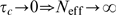, equation (20) converges to equation (15) with 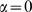 and 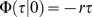. The error rate, 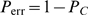, decays to zero exponentially with the number of cells, 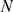. In the presence of correlations, 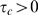, for small populations, 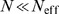, the tWTA error rate decays exponentially with 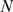, as in the uncorrelated case, equation (15). When 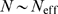, tWTA performance reaches the saturation regime, and tWTA accuracy converges to a finite limit for 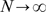:
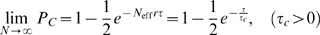
(22)Hence, in the presence of correlations for large 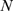, the tWTA error rate is an increasing function of 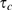, which saturates to chance level (chance level: 
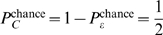
) in the limit of 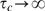.

#### Effect of baseline firing on tWTA accuracy

In the above analysis we assumed zero baseline firing for all cells. However, nonzero baseline firing may have a significant effect on the tWTA accuracy. To incorporate baseline firing into our model, it is most convenient to use the framework of the IHPP, which is defined by the PSTH. The PSTHs of the two populations are modeled by:
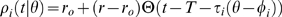
(23)where, 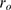 is the baseline firing rate (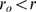) and 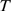 is the duration in which both columns fire at baseline prior to responding selectively to the stimulus. The function 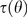 is the tuning of the delay parameter. As above we take 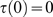 and 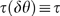. In this case, we find:
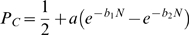
(24)

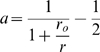
(25)

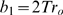
(26)

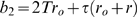
(27)


**Figure 4:**
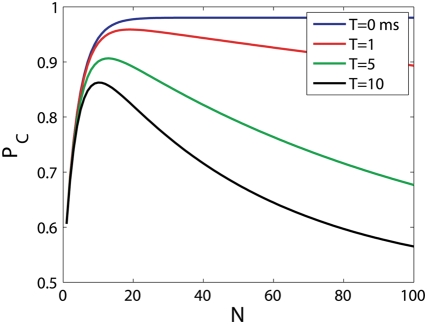
Effect of baseline firing on tWTA readout accuracy. The probability of tWTA correct response, 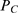, in the case of nonzero baseline firing is shown as a function of the population size, 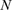, equation (24), for 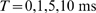 from top to bottom. Parameters used for this graph are: 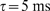, 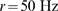 and 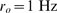.

Figure 4 shows the probability of correct discrimination as a function of 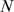 for different values of 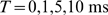 from top to bottom. For any positive 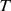, the probability of correct discrimination, 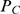, decays to chance level, 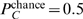, exponentially fast with 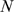 for large 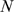. This decay results from the fact that the probability of not spiking in the time interval before time 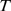 decays to zero exponentially with 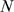. For 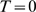, the probability of correct response will saturate exponentially to 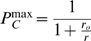 (compare with equation (9)) which can be high for low baseline firing rate, 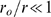. For small 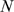, there exists a region, 
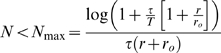
, in which 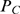 increases with 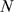.

#### The temporal *n* Winners-Take-All (*n*-tWTA)

**Figure 5:**
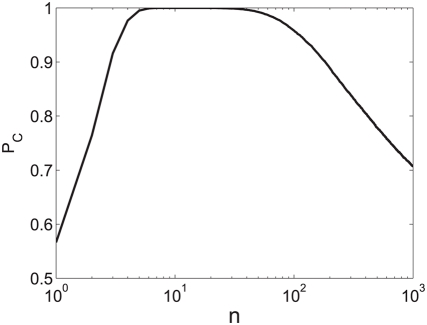
Performance of the 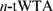 readout in a 2AFC discrimination task in a two-column model. The probability of correct discrimination of the 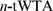 readout is shown as function of 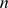. The probability of correct discrimination was estimated by averaging over 10^5^ realizations of the neural stochastic response in an IHPP model for spike time distribution as defined in equation (23) with: 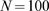, 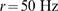, 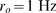, 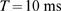 and 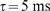.

To overcome the detrimental effect of baseline firing we generalize the tWTA to a family of readouts, 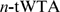, that are determined by the subgroup of cells that fired the first 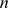 spikes. In a 2AFC competition between two homogeneous columns, the 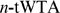 estimates the stimulus by the preferred direction of the column that fired the first 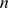 spikes. In the model of delayed step function response PSTH, equation (23), spikes that are fired in the absolute delay period, from time 0 to time 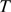, are independent of the stimulus and hence carry no information. The informative time of spiking is that from time 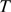 to time 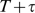, where firing rates of the cells depend on the stimulus. For a given population size, 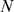, the mean number of spikes fired during the absolute delay time is 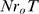. During the informative period, an average of 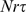 spikes is being fired by the informative group. Taking 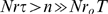 diminishes the detrimental effect of baseline firing and conserves the essential information embedded in the temporal order of the neural responses. Figure 5 shows the percent correct discrimination of the 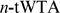, as a function of 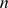. In this case, the average number of baseline spikes fired during the absolute delay time is 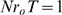, and 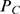 does indeed increase as 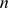 is increased from 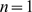 and to almost perfect discrimination at about 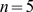. During the informative period of spiking, an average of 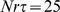 spikes are fired by the ‘correct’ group. As expected, the probability of correct discrimination deteriorates for 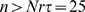. In this example, the performance of the 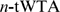 will decay to chance level in the limit of large 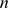, since we did not incorporate any scale differences in the firings of the two populations. Thus, a reasonable choice of 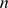 can eliminate the effect of baseline firing and greatly improve the performance of the tWTA.

Note that the optimal region for 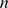, depends on the population size. For any fixed 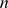, increasing the population size increases the number of baseline spikes fired during the absolute delay period, 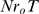. Hence, for 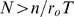 the 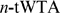 performance will decay to chance level. An alternative 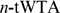 generalization is to estimate the stimulus by the preferred direction of the first *single* cell that fired 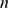 spikes, see [2]. Results for this later generalization are qualitatively similar to those of the former in this model.

### tWTA Estimation Accuracy in a Hypercolumn Model

#### The model

We study the tWTA estimation accuracy in a hypercolumn model of 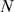 cells coding for an angular variable, 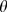, such as the coding for the direction of motion of a visual stimulus by MT cells. Each cell 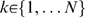 is characterized by its preferred direction 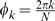 to which it responds fastest. Spike time distributions of different cells are modeled by independent IHPPs with PSTH 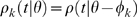, for cell 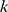, 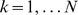.

The tWTA estimate of the stimulus is given by the preferred direction, 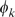, of the cell 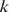 that fired the first spike
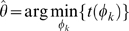
(28)where 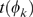 denotes the time of the first spike of cell 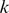, following presentation of the stimulus. Throughout this section, we quantify tWTA sensitivity by the inverse of the root-mean-square estimation error, 
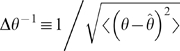
, where 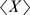 denotes averaging of 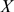 over the distribution of spike times for a given external stimulus 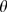.

#### tWTA accuracy in the absence of correlations

The probability of the tWTA estimator to be 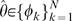, is given by the probability that the first spike in the population was fired by the cell with preferred direction 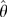:
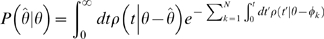
(29)


**Figure 6:**
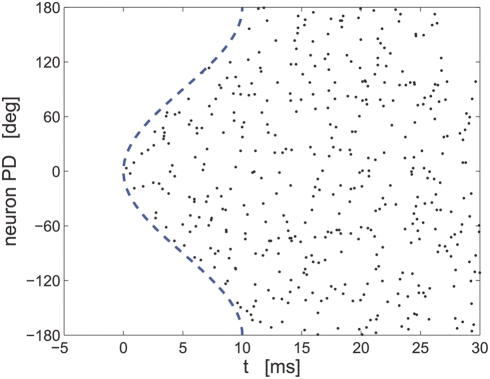
Simulation of a hypercolumn population raster plot. Spiking responses of 360 cells coding for an external stimulus 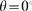 during a single trial are shown. Each line shows the spike-times of a single cell. The cells are arranged according to their preferred directions. Spike times of cell with preferred direction 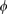 was modeled by an IHPP with PSTH 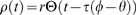, where 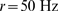 is the rate and the latency function is 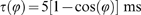. The dashed line shows 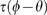.

Empirical examples of first spike time tuning to an angular external stimulus is shown for example in [1],[2]. Since tuning of the delay parameter makes the dominant contribution to the tWTA accuracy (see above), we now analyze the case of a delayed step function PSTH model with stimulus-independent scale and shape parameters. Specifically, we take the instantaneous firing rate of cell 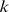 with preferred direction 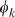, given that stimulus 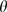 was presented at time 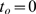, to be:
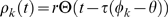
(30)This simple choice of PSTH does not limit the generality of our results but rather clarifies the analysis such that our conclusions are not obscured by non-relevant parameters. Figure 6 shows typical population response to stimulus 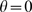. The dots on row 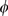 show the spike times of a single cell with preferred direction 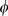. The dashed line shows the delay tuning function, 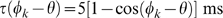, which yields the minimum possible spike time for each preferred direction.

The delay tuning function, 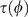, is assumed to be a periodic function of 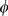. We further assume that the delay function, 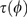, is a continuous, even function of its argument with a single minimum at 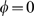. For cells with preferred directions close to the stimulus, we can approximate the delay function by:
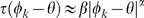
(31)where 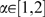 characterizes the delay tuning function near its unique minimum, for a smooth delay function 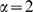, and 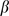 is a constant in units of time. Since the tWTA is affected only by the fastest cells, we can use the approximation of equation (31) to describe the delay function of the entire hypercolumn, bearing in mind that the likelihood of cells with preferred directions that are far removed from the stimulus to affect the tWTA decays exponentially fast with 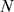.

**Figure 7:**
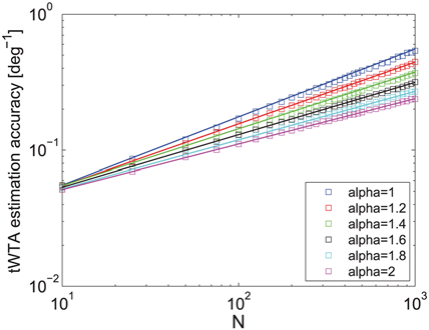
Estimation accuracy of the tWTA readout in a hypercolumn model. The accuracy of the tWTA readout, in terms of one over the squared estimation error of estimating 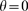, is plotted as a function of the population size, in an IHPP hypercolumn population model, equation(30). The latency tuning was modeled by 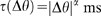 (where 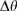 is measured in radian) with 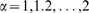 from top to bottom. Accuracy was measured by averaging the squared estimation error over 10,000 trials of simulating the neuronal stochastic response (squares). The solid lines show the analytical fit using equation (33).

Using the continuum limit approximation for the exponent in the right hand side of equation (29), we evaluate the conditional probability density of the estimation error of 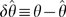 and obtain:
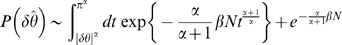
(32)In the limit of large 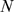, 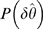 is of 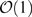 in 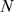 for 
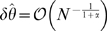
 and decays exponentially with 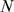 for 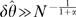. Hence, we obtain the following scaling law for the tWTA accuracy:
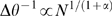
(33)As in the two-column competition in the 2AFC paradigm, the sensitivity of the tWTA readout in a hypercolumn model scales *algebraically* fast with 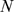, in the absence of noise correlations and in the limit of low baseline firing. This fast scaling is in contrast to the slow logarithmic scaling of the conventional rate-WTA readout accuracy wih the population size [24]. Figure 7 shows tWTA sensitivity, in terms of the inverse root mean square estimation error, as a function of the population size in a hypercolumn model for 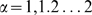 from top to bottom. The open squares show numerical estimation of the sensitivity as obtained by averaging tWTA error over 10^4^ realizations of simulating the network stochastic response. The solid lines show fits using the analytical result of equation (33) with 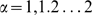 from top to bottom.

#### Effect of correlations on the tWTA estimation accuracy

**Figure 8:**
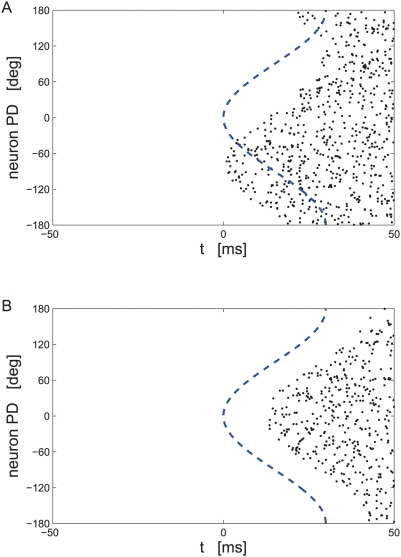
Simulation of a hypercolumn population raster in the presence of correlations. Spiking responses of 360 cells coding for an external stimulus 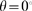 during a single trial are shown. Every line shows the spike-times of a single cell. The cells are arranged according to their preferred directions. Spike times are distributed as defined in the section ‘effect of correlations on tWTA accuracy’, see equation (34), with 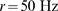 and 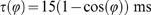. For the correlated part: (a) 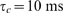, 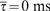; (b) 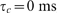, 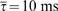. The dashed line shows 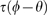.

To model first spike time correlations in a hypercolumn, we write the spike times of each cell as the sum of correlated and uncorrelated parts
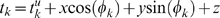
(34)where the uncorrelated parts, 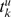, are taken to be distributed according to an IHPP with a PSTH 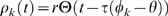. For the sake of simplicity, we take 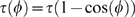. The terms 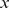, 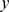 and 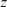 are the correlated components of the spike times. The 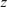 term represent the effect of shared input to the entire hypercolumn, whereas, 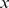 and 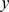 represent the effect of shared input that is stronger for columns that are functionally closer, i.e., have smaller preferred directions difference. We assume that the correlated noise has zero means 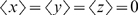 and variance 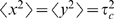 and 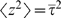. Figure 8 shows typical realizations of the population response during a single trial of presenting stimulus 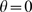 in the presence of noise correlations. In Figure 8b 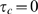 and 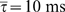. The uniform correlations generates collective fluctuations that shift the entire population response right (as in the specific realization in the figure) and left of the dashed line that shows 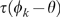. Nevertheless, this fluctuation exists in a collective mode of the neural responses that does not alter the order of firing and hence does not affect tWTA accuracy. In Figure 8a 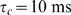 and 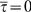. In this case, the collective fluctuations shift the response of the entire population up and down (as in the specific realization in the figure). These fluctuations limit the accuracy in which the tWTA can estimate the stimulus. In the limit of large 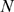, the error is dominated by the correlated response. Neglecting the uncorrelated part of the fluctuations, we obtain (see Methods):
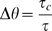
(35)where 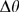 is measured in radians. Note that equation (35) takes the form of a signal-to-noise ratio, where the signal is the modulation amplitude of the delay function, 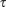, and the noise is the component of collective noise correlations that affect the tWTA estimation, 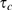. The tWTA sensitivity, equation (35), is independent of the collective fluctuations in the uniform direction, 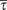.

**Figure 9:**
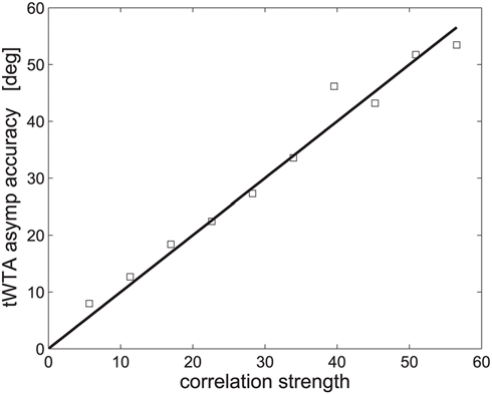
Effect of correlations on the asymptotic tWTA estimation accuracy in a hypercolumn model. tWTA accuracy, in terms of the root mean square estimation error, 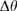, is shown as a function of the correlations' strength, 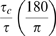, in a hypercolumn model, as defined in section ‘effect of correlations on the tWTA estimation accuracy’, see equation (34). The solid line shows the analytical asymptotic value, equation (35). Open squares show the numerical estimation of the asymptotic value as calculated by averaging the tWTA estimation error over 100 trials in a hypercolumn model of 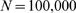 cells. The latency function that was used was: 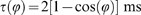. To minimize the effect of finite 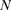, an extremely high firing rate of 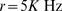 was used in the IHPP simulations.

Figure 9 shows the asymptotic accuracy of the tWTA as a function of the noise-to-signal ratio 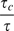. The solid line shows the analytical result of equation (35) in the limit of large 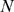. The open squares show numerical estimation of asymptotic accuracy using a population of size 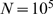 cells. The finite size of the network limits the ability of the numerical estimate to follow the analytic curve at high accuracy (low noise levels). To compensate somewhat for this effect, an extremely high firing rate was used in the simulations.

#### Effect of baseline firing on the tWTA accuracy

**Figure 10:**
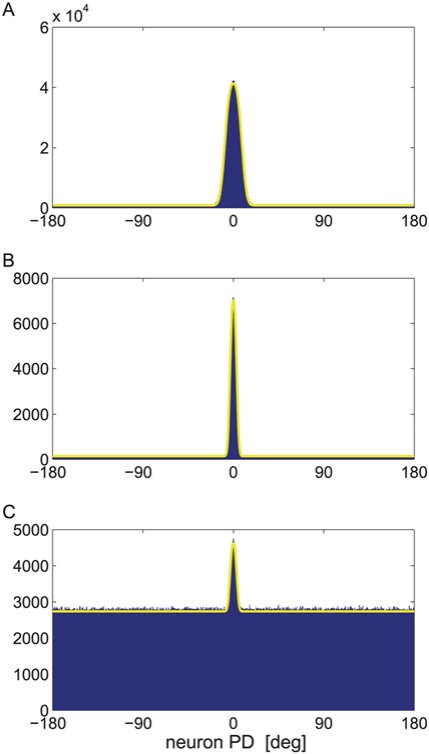
Effect of baseline firing on the tWTA estimation in a hypercolumn model. Histograms of tWTA estimation of stimulus 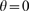 were obtained in a model of delayed step function response to the stimulus, equation (36), with 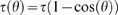, and parameters: 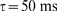, 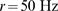 and 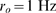. Population size was 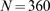 in (a) and 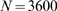 in (b,c). Histograms were estimated using 10^6^ repetitions in (a,b) and using 10^7^ repetitions in (c). The solid lines are analytical approximations of equation (37) in (a,b) and equation (38) in (c).

The effect of nonzero baseline firing on tWTA estimation accuracy is studied in the framework of a hypercolumn IHPP model with a delayed step function PSTH. Specifically, we took the following PSTH for the response of cell 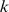 with preferred direction 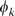:
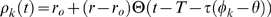
(36)where 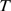 is the absolute delay, 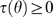 is the tuning of the delay parameter with 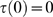. For 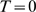 in the limit of large 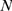, we can approximate the probability of the tWTA estimator to be 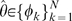, equation (29), by:
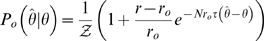
(37)where 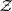 is a normalizing factor of the probability distribution. Figure 10a and 10b show histograms of tWTA estimations of stimulus 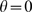 for 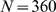 and 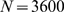, respectively, in this model with 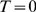. The solid line shows the analytical approximation, equation (37). The distribution is characterized by a narrow peak around zero error, with a width that decreases to zero as 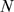 grows to infinity and a uniform probability for large errors. The ratio of the peak distribution of the zero error (at 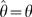) to the distribution of a specific large error is given by 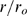. However, since the width of the peak decreases as 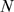 increases (compare Figure 10a and 10b), the average squared estimation error increases for large 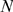, even in for 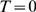, in contrast to the effect of baseline firing in the 2AFC, where at 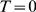 the probability of correct response is an increasing function of 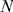. A hallmark of the tWTA readout is the high kurtosis of the estimation error.

In the case of 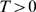, using equation (37), one obtains
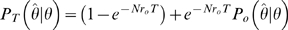
(38)Hence, in this case the peak to base ratio of the distribution is decreased and decays exponentially to zero with the product 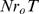. This effect is shown by the histogram of tWTA estimation errors in Figure 10c where we took 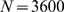 and 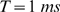 (compare with Figure 10b where 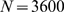 and 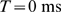). The solid line shows the analytical approximation of equation (38).

## Discussion

At the time of the first spike, the tWTA is the ideal observer and, in the case of angle estimation, it is also the population vector readout. If a decision must be made at very short times, then the tWTA is the best readout. It is therefore important that we know and understand the capabilities and limitation of this readout. Scaling of the tWTA accuracy with the population size, 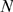, can show a wide range of behaviors: from constant in 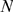 (equation 9), through logarithmic (equation 13) to algebraic (equation 17). These different scaling regimes depend on fine details of the tuning of the probability distribution of the first-spike-times or alternatively on the initial rise of the PSTH response to the stimulus. In the generic case in which scale, shape and delay parameters are all tuned to the stimulus, the tWTA accuracy will increase algebraically with 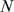, in contrast to the expected logarithmic slow scaling of the conventional rate-WTA readout [24]. Nevertheless, the tWTA is expected to show high sensitivity to the inherent neuronal diversity at the level of single cell response properties (see e.g., [30]). This sensitivity of the tWTA predicts considerable subject-to-subject variability in psychophysical performance as well as large fluctuations in the psychophysical accuracy for the same subject under different stimuli conditions, such as discriminating 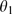 and 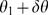 versus discriminating 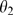 and 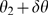.

Noise correlations in the fluctuations of first-spike times of different cells have a drastically detrimental effect on the tWTA accuracy, limiting the effective number of degrees of freedom in the network and resulting in finite error levels, even in the limit of large 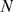, see e.g., equations (21), (22) and (35) and Figures 3 and 9. This effect is similar to that has been reported in population coding literature [25]–[29],[31], and depends on the correlations structure. Here we investigated the effect of correlations that had simple spatial structure and no temporal structure. A drastically detrimental effect on the tWTA accuracy is caused by neuronal response covariance which generates collective fluctuation that resembles the ‘signal’, i.e., similar to the tuning of the delay parameter (see Figure 8). For a detailed discussion on the effects of correlations structure see [27]. The temporal structure of response covariance may also have a considerable effect. For example, if the correlations depend on the absolute time, in a manner that they are negligible for small 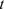 and build up later in time, then they will not necessarily cause saturation of the tWTA accuracy. However, better empirical understanding of first spike time correlations is required to yield sufficient constraint for theoretical study of this issue. It is important to emphasize that by correlations we mean first spike time covariance of *simultaneously* recorded cells, in contrast to other types of correlations [5].

In a 2AFC, task nonzero baseline firing has a twofold detrimental effect on the tWTA accuracy. The first is in the case in which the onset of the tWTA readout precedes the stimulus response of the fastest cell in the entire population, 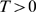. In this case, the tWTA accuracy will decrease as 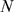 is increased beyond some optimal value 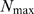. This effect can be minimized by obtaining a more accurate estimate for the minimal response time of the cells in the population, i.e., effectively decreasing 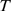 [5]. The second effect is a saturating effect, which limits the maximal accuracy that can be obtained by the tWTA, 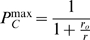, even for 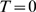. Note that, although 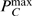 is less than 1, psychophysical accuracy is also finite. The value of 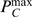 can be rather high in cases in which the baseline firing is small relative to the stimulus response. These effects can be decreased for any given 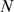 by using a generalized 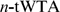 readout that makes a decision according to the population that fired the first *n* spikes, see Figure 5. Nevertheless, for any given fixed value of 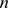, increasing the population size, 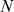, will decrease the 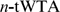 performance to chance level, for 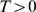. Hence, for fast decisions there are advantages to reading out the responses of small neuronal populations rather than larger populations.

Baseline firing has similar detrimental effects on the tWTA readout in estimation tasks (see Figure 10). A hallmark of the tWTA readout that can serve as a prediction is its high kurtosis. There are various ways to generalize the tWTA to use more than one spike in order to overcome the detrimental effect of baseline firing. One option is that readout is determined by the preferred direction of the single cell that fired the first 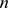 spikes. An alternative generalization is to define the readout by a ‘vote’ of cells that fired the first 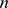 spikes in the population. In the later case, different weights may be assigned to the votes. The utility of the different possible generalizations is expected to depend largely on the structure of the correlations in the neuronal initial dynamic response to the stimulus.

In a series of highly influential papers, Thorpe and colleagues (see e.g., [12],[14]), have highlighted the possible role of spike latency as primary source of information in the CNS and have shown, for example, how an image falling on the retina could be reconstructed from a spike latency (see also work of [15]). In the context of this work, their readout could be thought of as a specific choice for the 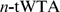 generalization. Here, we presented a systematic investigation of the tWTA accuracy that allows for comparison with psychophysical accuracy; hence, enables testing of the hypothesis that tWTA is actually used by the CNS. In addition, our analysis provides a framework that allows for the understanding and the investigation of the effects of correlations and baseline firing on the tWTA accuracy.

### 

#### Neural network implementations of the tWTA

Considerable theoretical effort has been devoted to the investigation of neural network models that can implement the conventional rate-WTA [32]–[43]. These studies have focused on inputs that are constant in time and differ by their scale. However, it has been acknowledged that the temporal structure of the inputs may have a considerable effect on the WTA readout [43]. This effect shows the sensitivity of existing rate-WTA neural network models to the order of firing and demonstrates the capability of neural networks to implement a tWTA computation. Indeed one can imagine the responses of the (assumed excitatory) hypercolumn network that code for the external stimulus by their spike time latency, being input to a 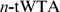 readout layer of laterally all to all connected inhibitory neurons. Once, input to a inhibitory cell crosses firing threshold of 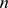 excitatory post synaptic potential, it will fire and silence the rest of the network. Investigation of various neural network implementations, their limitations and deviations from the mathematically ideal tWTA and the computational consequences of these deviations if exist is beyond the scope of the current work and will be addressed elsewhere.

#### The neural code

To what extent does the CNS use the tWTA as a readout mechanism? Readout mechanisms used by the CNS are necessarily dynamic processes that may involve inhibition and hence generate WTA-like competition between inputs from different columns. If fast decisions between a small number of alternatives are required, then the tWTA can provide the correct result with high probability. In such a case, we predict that the readout will be determined by competition between relatively *small* groups of cells rather than by the entire cell population that responds to the stimulus so as to decrease the effect of baseline firing. Such decisions include, for example, estimation of the direction of motion of a visual stimulus at a low resolution of 45°. However, for discrimination between many alternatives the tWTA is limited by the baseline firing. Why is this task more sensitive to baseline firing? Consider an example in which estimation of the direction of motion of a visual stimulus is required at a precision of 3.6°. For this angular resolution, a population of at least 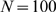 cells is needed. Let us assume that at the stimulus onset the ‘correct’ cell fires at a rate of 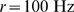 while the rest of the population fires at a baseline rate of 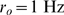. During the first 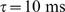 of stimulus presentation, the ‘correct’ cell will fire an average of 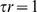 spike, while the rest of the cells will fire an average of 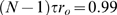 spikes; thus, the tWTA is expected to err in more than 3.6° in about 50% of the cases. Hence, fine estimation tasks cannot rely on the *single* first spike, and our theory predicts that in these cases the first 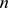 spikes must be considered where 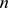 should be larger than the average number of baseline spikes. How should the 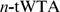 combine the information from the first 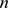 spikes? The answer to this question depends on the temporal structure of correlations, fine details of the PSTH, and on our assumptions on the computational capabilities of this readout and is beyond the scope of the current paper. The current work provides the essential framework for addressing this question. To further study the hypothesis that the CNS actually uses the tWTA better empirical understanding of the tuning of first spike time distribution to the stimulus, baseline firing, and the spatial and temporal structure of noise correlations is required.

## Methods

### Calculation of tWTA Accuracy in 2AFC in the Case of Delay Parameter Tuning to the Stimulus

Substituting equation (14) into equation (4), we obtain the probability of correct discrimination as sum of two terms:
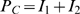
(39)

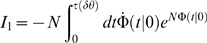
(40)

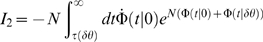
(41)The integral 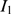, equation (40), can be evaluated exactly, yielding the contribution of
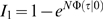
(42)where we have used 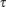 as shorthand for 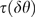. The contribution of 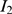 to 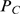 is positive; hence, the tWTA error rate, in this case, will decay to zero *exponentially* with the population size 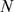.

For the calculation of 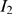, equation (41), we change variables to 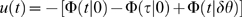, yielding:
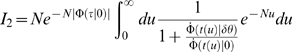
(43)where for a small positive 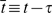, 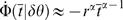. Assuming 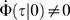, the leading term in 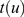, for small 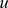, is given by:
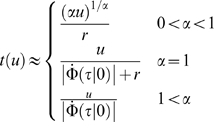
(44)Using Watson's Lemma to evaluate 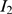 to leading order in 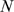, we obtain
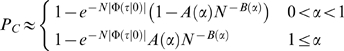
(45)where
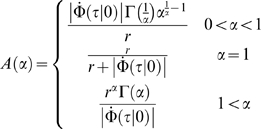
(46)and
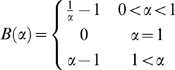
(47)


### Calculation of the tWTA Accuracy in 2AFC with Correlations

For a given stimulus 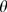, the probability density, 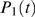, that the first spike of population 1 occurred at time 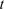 is
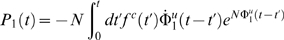
(48)where 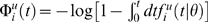. The probability density, 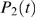, that population 2 did not fire before time 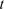 is given by:
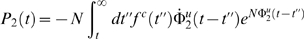
(49)Assuming the tWTA decides the stimulus is 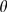 if the first spike comes from population 1, the probability of correct response is:
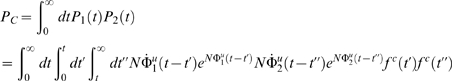
(50)In the limit of large 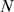, 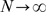, we obtain 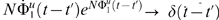, and due to the delay 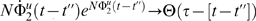, we thus obtain
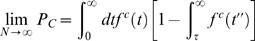
(51)


In the specific example of Figure 3, the following model was used. The uncorrelated part of the first spike times was generated by an IHPP with a delayed step function PSTH, yielding the first-spike-time probability density: 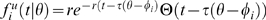 with 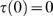 and 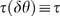. For the correlated part, we used an IHPP model with PSTH, yielding: 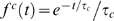. From equation (48), the probability density, 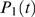, that the first spike in column 1 occurred at time 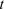, is given by:
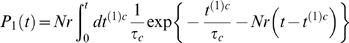
(52)The probability density, 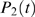, that no cell in column 2 had fired until time 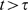, equation (49), is equal to the probability that the first spike of the cells in column 2 occurred at any time 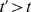:
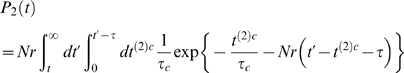
(53)Note that for 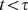, 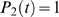. The probability of correct classification is given by:
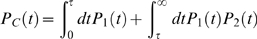
(54)Substituting equations (52) and (53) into equation (54) and integrating one obtains the result of equation (20).

### tWTA Accuracy in a Correlated Hypercolumn Model

We now turn to calculate tWTA asymptotic accuracy in the presence of correlations, see section ‘Effect of correlations on the tWTA estimation accuracy’. In the limit of large 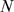, the estimation error will be dominated by the correlated noise. We can, therefore, neglect the fluctuations of the uncorrelated part, 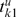 (see equation (34)), replacing its distribution with a delta function at the first time the PSTH of cell 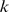 is larger than zero:
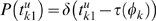
(55)In this case, for a specific realization of 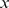, 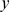 and 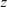 we can write the first spike time of cell 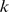 as
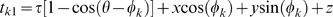
(56)Without loss of generality, we will assume 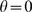. The tWTA estimate for the stimulus, 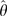 is obtained by taking the derivative of equation (56) with respect to 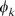 and equating to zero at 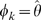


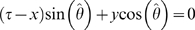
(57)For small errors, we can approximate:
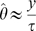
(58)and obtain:
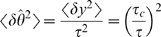
(59)which is equivalent to the result of equation (35).
